# Radiomic and dosiomic machine learning models for predicting radiation-induced oral mucositis in head and neck cancer: a systematic review

**DOI:** 10.1007/s12094-026-04427-0

**Published:** 2026-06-08

**Authors:** Lukas Mendes de Abreu, Raquel Molina Sanches, Alana Crescencio de Oliveira, Michele Di Benedetto, Izabel Regina Fischer Rubira-Bullen, Kellen Cristine Tjioe, Cássia Maria Fischer Rubira

**Affiliations:** 1 Department of Surgery, Stomatology, Pathology, and Radiology, Bauru School of Dentistry, University of São Paulo, Alameda Octávio Pinheiro Brisolla, 9-75, Bauru, SP 17012-901 Brazil; 2 Georgia Cancer Center, Medical College of Georgia, Augusta University, Augusta, GA USA

**Keywords:** Oral mucositis, Head and neck cancer, Radiomics, Dosiomics, Machine learning, Predictive modeling

## Abstract

**Objective:**

To critically evaluate machine learning (ML) models developed for predicting radiation-induced oral mucositis (OM) in head and neck cancer (HNC) patients, emphasizing the contribution of advanced imaging biomarkers such as radiomic and dosiomic features.

**Materials and methods:**

This systematic review followed the PRISMA 2020 guidelines and was registered in PROSPERO (CRD420251075245). The PICOS framework guided the review question and inclusion criteria. Studies developing or validating ML models for OM prediction using radiomic and/or dosiomic data were included. Electronic databases (PubMed, EMBASE, Web of Science, Scopus) were searched up to July 2025. The risk of bias was assessed using the PROBAST tool, and models were compared based on predictor types.

**Results:**

Seven studies involving 2,311 patients were included. All models incorporated dosimetric predictors, while some used additional radiomic (n = 3), dosiomic (n = 2), contouromic (n = 1), or oral health indices (n = 1). Reported discrimination (AUC) ranged from 0.70 to 0.96, but external validations showed reduced performance (AUC ~ 0.65). High risk of bias was mainly observed in analysis domains due to limited sample sizes and incomplete calibration.

**Conclusion:**

ML models for OM prediction remain exploratory and are not yet ready for routine clinical implementation due to poor generalizability and calibration issues.

**Supplementary Information:**

The online version contains supplementary material available at https://doi.org/10.1007/s12094-026-04427-0.

## Introduction

Radiation-induced oral mucositis (OM) is a severe acute toxicity of radiotherapy (RT) for head and neck cancer (HNC), with incidence rates reaching 80–100% in patients receiving concurrent chemoradiotherapy [1, 2]. This inflammatory reaction manifests as painful ulcerations in the oral cavity, often leading to dysphagia, weight loss, and treatment interruptions that compromise tumor control [1, 2]. Beyond clinical morbidity, OM substantially increases healthcare utilization and treatment costs, underscoring its significance as both a medical and economic burden [3].

Traditional prediction models for OM have largely relied on dose–volume histogram (DVH) parameters and selected clinical covariates [4–6]. Although these normal tissue complication probability (NTCP) models form the foundation of the field, they explain only part of the clinical variability, leaving a considerable proportion of individual patient risk unaccounted for [6]. Attempts to refine these models by adding variables such as age, sex, or tumor stage have yielded inconsistent findings, underscoring the limitations of purely dosimetric–clinical approaches [4, 5].

The advent of machine learning and advanced quantitative imaging has introduced radiomics and dosiomics as potential avenues for toxicity prediction [7–9]. Radiomics extracts high-dimensional quantitative descriptors from routine medical images, uncovering sub-visual phenotypes, while dosiomics captures spatial heterogeneity in three-dimensional dose distributions beyond conventional DVHs [7–9]. Dosiomics is strictly defined as the extraction of spatial heterogeneity and texture features from three-dimensional dose distributions [8]. This distinguishes it from simpler mathematical transformations of standard dose-volume histograms, such as principal component analysis, which do not inherently capture spatial dose topology [8]. When integrated with clinical features, these multi-omic pipelines offer the potential to build more accurate and generalizable predictive models for radiation-induced toxicities [7–9].

Although pioneering studies have extended these approaches to OM, the evidence remains preliminary and fragmented. Dean et al. [10] introduced spatial dose metrics and machine learning for NTCP modeling of OM, while Musha et al. [11] developed an oral mucosal dose-surface model in carbon-ion radiotherapy. Despite their innovation, these efforts lacked harmonization, external validation, and systematic synthesis. Addressing this gap, the present systematic review critically evaluated the methodology, performance, and validity of radiomic- and dosiomic-based prediction models for OM, aiming to consolidate the current evidence base and inform future research toward clinically translatable solutions.

## Materials and methods

This systematic review was conducted in accordance with the Preferred Reporting Items for Systematic Reviews and Meta-Analyses (PRISMA) 2020 guidelines [12]. The protocol was prospectively registered in the International Prospective Register of Systematic Reviews (PROSPERO) (registration number: CRD420251075245).

The review was designed to address the following focused research question: *“Are radiomics or dosiomics, when combined with machine learning methods, effective in predicting radiation-induced oral mucositis in patients with head and neck cancer?”* The question was structured using the PICOS framework, as follows: 1. Population, adults (18 years or older) diagnosed with HNC, including nasopharyngeal carcinoma (NPC); 2. Intervention, RT, with or without concurrent chemotherapy, in which machine learning was applied for predictive model development; 3. Comparison, not applicable, as this was a synthesis of predictive modeling studies; 4. Outcome, radiation-induced OM; and 5. Study design, including prospective and retrospective cohort studies.

### Information sources and search strategy

A comprehensive search strategy was applied across four electronic databases: PubMed, EMBASE, Web of Science, and Scopus. Searches were conducted from database inception through July 2025, with no restrictions on publication year or language. The full electronic search strategies for each database are provided in Supplementary Table 1.

To ensure completeness, the reference lists of all eligible studies were manually screened to identify additional publications not captured by the electronic searches. Abstracts, conference proceedings, editorials, case reports, and review articles were excluded.

### Study selection and eligibility criteria

All retrieved records were imported into Rayyan [13], a web-based platform that facilitates blinded screening and conflict resolution in systematic reviews. Two reviewers independently screened titles and abstracts to identify potentially eligible studies, and discrepancies were resolved through discussion or consultation with a third reviewer. Full texts of studies deemed potentially eligible were then assessed independently by the same reviewers. Studies were included if they employed a prospective or retrospective cohort design, developed or validated a prediction model for radiation-induced OM, and utilized radiomic and/or dosiomic analyses applied to imaging (e.g., planning CT) or dose-related data. Studies were excluded if they only assessed associations between clinical variables and OM without model development, did not involve radiomic or dosiomic features, or were case reports, reviews, editorials, or conference abstracts lacking sufficient data for extraction.

### Data extraction and synthesis

Two reviewers independently extracted data using a standardized and piloted form. Extracted variables included: study characteristics (authors, year, country, study center, sample size), patient demographics (age, sex), treatment modalities, mucositis definitions and grading scales, imaging modalities, feature categories (clinical, dosimetric, radiomic, contouromic, or oral health–related), machine learning algorithms, validation strategies, and performance metrics including the area under the receiver operating characteristic curve (AUC), accuracy, sensitivity, and specificity. Owing to methodological and endpoint heterogeneity, a quantitative synthesis was deemed inappropriate.

Given the methodological heterogeneity across studies (in endpoints, imaging, algorithms, and validation strategies), a formal meta-analysis was not feasible. Additionally, the performance of the proposed multi-omic models was qualitatively compared against their respective clinical or dosimetric baselines to evaluate their incremental predictive value.

### Risk of bias and applicability assessment

Risk of bias (RoB) and concerns regarding applicability were independently evaluated by two reviewers using the Prediction Model Study Risk of Bias Assessment Tool (PROBAST) [14, 15]. The four PROBAST domains Participants, Predictors, Outcome, and Analysis were each rated as low, high, or unclear risk of bias, based on responses to signaling questions.

A rating of low risk was assigned when all criteria within a domain were satisfied, indicating methodological rigor. A high-risk rating was given if at least one critical flaw was identified, such as lack of external validation, inadequate sample size, or insufficient handling of missing data. When the available information was insufficient to make a judgment, the risk was rated as unclear. Applicability was assessed by examining alignment of the study population, predictors, and outcomes with the research question. Discrepancies between reviewers were resolved by consensus.

## Results

### Study selection

**Fig. 1:**
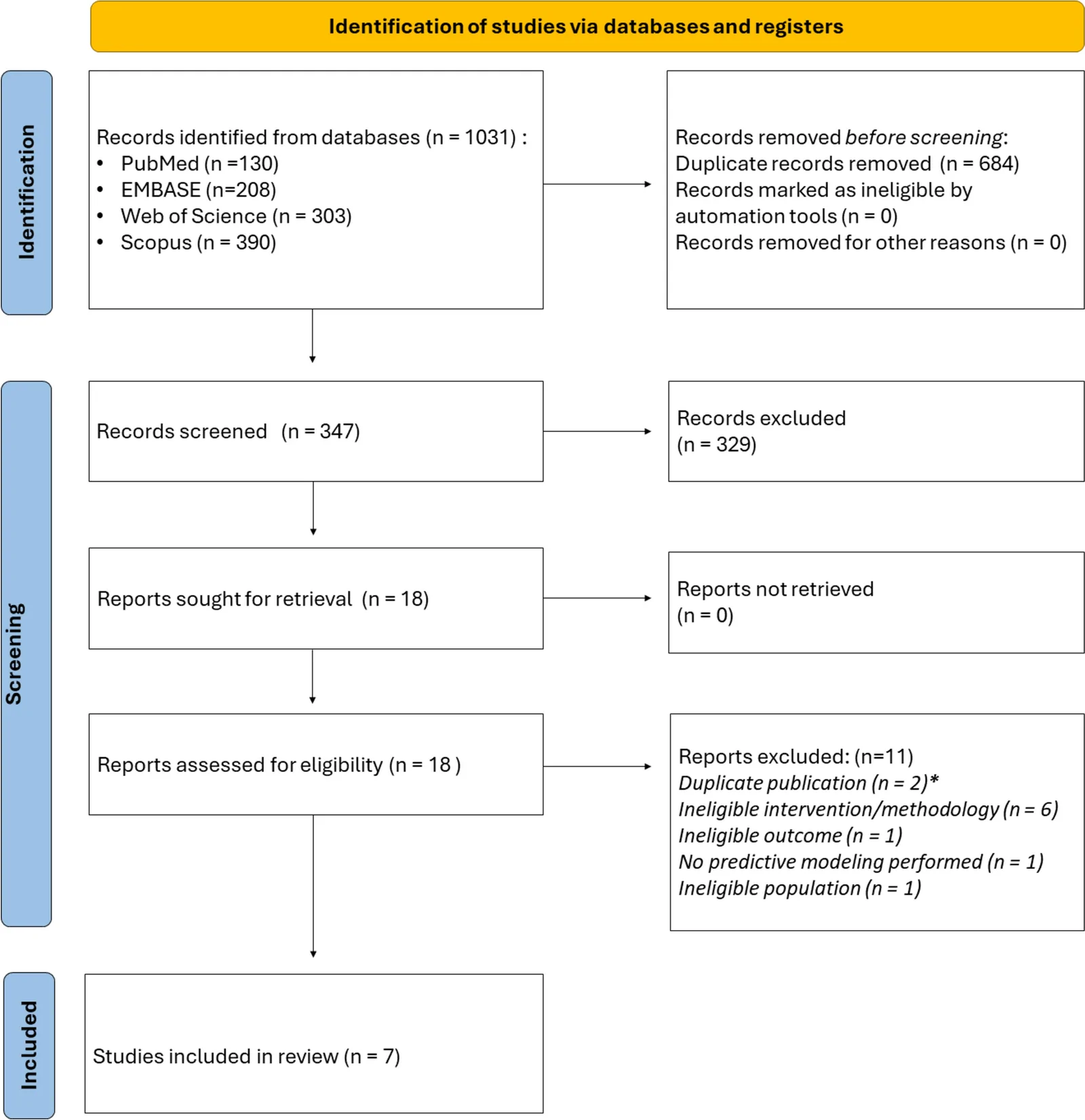
PRISMA 2020 flow diagram of study selection for the systematic review. * The study reports on a patient cohort that is also included in another study

A total of 1,031 records were retrieved from the database search. After the removal of 684 duplicate records, 347 records remained for screening based on titles and abstracts. Of these, 329 records were excluded, leaving 18 reports for full-text review. All 18 full-text articles were assessed for eligibility. Of these, 11 were excluded for various reasons, as detailed in Supplementary Table 2. These reasons included: duplicate publication (n = 2), ineligible intervention/methodology (n = 6), ineligible outcome (n = 1), no predictive modeling performed (n = 1), and ineligible population (n = 1). Ultimately, 7 studies met all eligibility criteria and were included in the final synthesis. The study selection process is summarized in the PRISMA flow diagram (Fig. 1).

### Study characteristics

The seven included studies comprised a total of 2,311 patients treated for HNC or NPC with radiotherapy, with or without concurrent chemotherapy. Cohort sizes varied substantially, from small exploratory single-center series of fewer than 50 patients [19] to large institutional registries with more than 800 participants [17], including two multicenter studies [16, 20]. Geographically, studies spanned the United Kingdom [16], Denmark [17], Japan [18, 21], Iran [19], and China [20, 22].

RT modalities were also heterogeneous: most cohorts received intensity-modulated radiation therapy (IMRT) or volumetric-modulated arc therapy (VMAT) [16–20, 22], whereas one study investigated carbon-ion radiotherapy (CIRT) [21]. Chemotherapy regimens varied, with several studies reporting concurrent cisplatin as standard of care [18, 22].

Most studies relied on the Common Terminology Criteria for Adverse Events (CTCAE), with versions ranging from v2.0 to v5.0 and thresholds typically set at grade ≥ 3, except for Rachi (2023) [18], who considered grade ≥ 2 requiring adaptive RT as the clinically relevant outcome. Endpoint definitions exhibited non-trivial heterogeneity across studies, constituting a major limitation for direct cross-study comparison. This heterogeneity in endpoint definition complicates cross-study comparability and may contribute to performance variability. A detailed summary of study characteristics, predictive features, main findings, and limitations is provided in Table 1, with an expanded synthesis of key findings presented in Supplementary Table 3.

**Table 1:** General characteristics of included studies

Study	Country/Study Center	Cancer type	No. of patients (training/validation)	Median Age in years (range)	Male/Female	Treatment received	Oral mucositis grading scale
Dean et al., 2017	United Kingdom/Multi-center	HNC	179 (NR/NR)	57 (17–88)	114/65	RT (different doses and fractionations); some patients received concurrent chemotherapy	CTCAE v2.0 or v3.0 Severe OM: Grade ≥ 3
Hansen et al., 2020	Denmark/Single-center	HNC	802 (535/267)	62 (31–86)	600/202	IMRT (predominantly VMAT), with or without concurrent chemotherapy	DAHANCA mucositis scale (equivalent to CTCAE v3.0) Severe OM: Grade ≥ 3
Rachi et al., 2023	Japan/Single-center	HNC	61 (NR/NR)	NR	NR	Concurrent CRT with cisplatin and VMAT	CTCAE v5.0 (Grade ≥ 2)
Agheli et al., 2024	Iran/Single-center	HNC	49 (39/10)	52 (22–81)	36/13	IMRT	CTCAE v5.0 (Grade ≥ 3)
Nicol et al., 2025	China/Multi-center	NPC	464 (363/101)	DC: 54 (NR) VC: 57 (NR)	347/117	RT, with concurrent CRT as standard of care	CTCAE v5.0 Severe OM: Grade ≥ 3
Meng et al., 2025	Japan/Single-center	HNC	178 (124/54)	64 (52–74)	87/91	CIRT	CTCAE v4.0 Acute OM: Grade > 2
Zeng et al., 2025	China/Single-center	NPC	578 (430/148)	46 (17–81)	443/135	IMRT with concurrent chemotherapy	CTCAE v5.0 Severe OM: Grade ≥ 3

*HNC* Head and neck cancer; *NPC* Nasopharyngeal carcinoma; *NR* Not reported; *DC* development cohort; *VC* validation cohort; *RT* Radiotherapy; *CRT* Chemotherapy; *IMRT* Intensity-modulated radiation therapy; *VMA* Volumetric modulated arc therapy; *CIRT* Carbon-ion radiotherapy; Version 2.0, *CTCAE v2.0* common terminology criteria for adverse events; *CTCAE v3.0* common terminology criteria for adverse events, Version 3.0; *CTCAE v4.0* common terminology criteria for adverse events version 4.0; *CTCAE v5.0* common terminology criteria for adverse events, version 5.0; *DAHANCA* Danish head and neck cancer group

### Predictive features and modeling approaches

Across the seven studies, all models incorporated dosimetric information, most frequently dose–volume histogram (DVH) parameters or spatial dose descriptors, complemented by sociodemographic and clinical covariates such as age, sex, and treatment factors. Beyond this shared foundation, each study adopted distinct strategies to expand predictive capacity. Dean et al. (2017) [16] pioneered the use of three-dimensional moment invariants to capture spatial dose distributions and compared a novel oral mucosal surface contour (MSC-PM) against the conventional oral cavity planning margin (OCC-PM). Hansen et al. (2020) [17] applied principal component analysis (PCA) to the DVHs, thereby reducing collinearity and generating independent dose components (PC1–PC2). This approach was coupled with treatment acceleration, smoking status, and sex as additional clinical covariates.

In contrast, smaller single-center studies adopted more exploratory strategies, often with highly specific predictor sets. Rachi et al. (2023) [18] focused exclusively on pre-planning parameters, generating 671 five-dimensional predictor lists from just 61 patients. The most influential features included equivalent square irradiation field (EQIF) size, total monitor units, dose per segment, CTV high-to-BMI ratio, and mean oral cavity dose. Agheli et al. (2024) [19] integrated radiomic, dosimetric, and clinical data, beginning with nearly one thousand features (851 radiomic, 84 dosimetric, and 38 clinical), which were narrowed to a final panel of 20 predictors through minimum redundancy–maximum relevance (mRMR) selection.

**Fig. 2:**
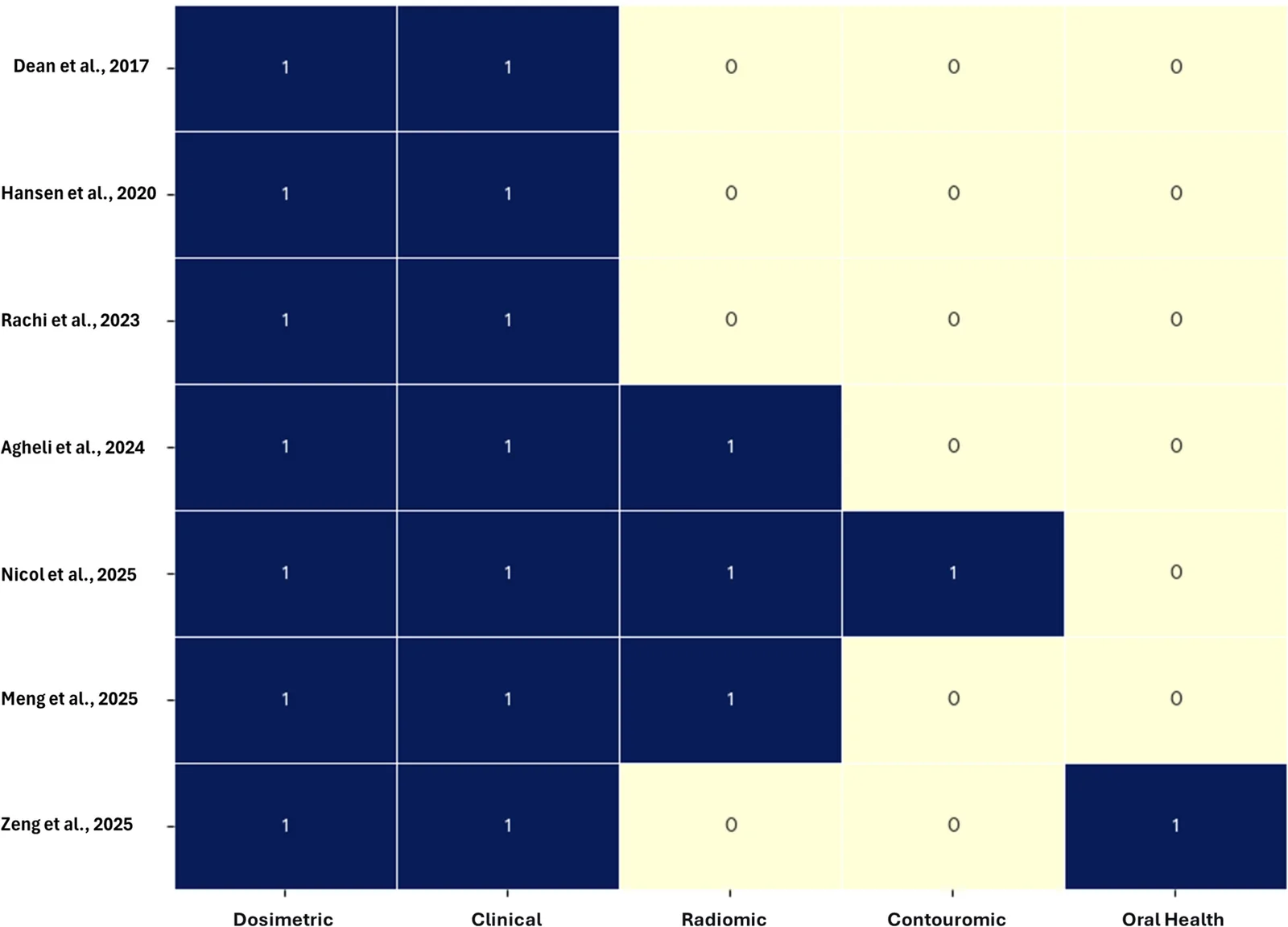
Summary of predictive modeling approaches for radiation-induced oral mucositis, from dosimetric–clinical to multi-omic models

Multimodal integration was further expanded in multicenter and modality-specific contexts. Nicol et al. (2025) [20] began with 2,206 features spanning clinical, radiomic, dosiomic, and contouromic domains, ultimately reducing them to five (one clinical, three radiomic, one dosiomic) for a parsimonious random forest model. Meng et al. (2025) [21], focusing on CIRT, developed a hybrid NTCP model with three complementary predictors: a DVH parameter (V37 Gy[RBE]), one radiomic descriptor (GLCM_MCC), and one dosiomic feature (GLSZM_HGLZE). Finally, Zeng et al. (2025) [22] incorporated host-related oral health indices, namely the Dental Calculus Index (DCI) and Oral Cleanliness Index (OCI), in combination with conventional dosimetric measures, marking the first attempt to integrate oral hygiene into OM prediction. These approaches highlight the gradual progression from simple dosimetric–clinical models to increasingly complex multi-omic pipelines (Fig. 2; Table 2).

**Table 2:** Imaging modalities, predictors, and modelling approaches

Study	Imaging modality	Radiomic/Dosiomic features	Additional predictors	ML algorithm(s)	Validation strategy
Dean et al., 2017	Planning CT (OAR contouring)	DVH parameters; 3D dose moment invariants (ηabc)	Clinical: age, sex, chemotherapy, tumor site	Penalized logistic regression (PLR), Random Forest (RF)	Internal, 100-iteration stratified shuffle split (80/20); no external validation
Hansen et al., 2020	Planning CT	DVH parameters (oral cavity); reduced via PCA (5 components)	Clinical: sex, chemotherapy (weekly low-dose), treatment acceleration, age, smoking status	LASSO, Best Subset Selection	Internal: 2/3 development, 1/3 validation; tenfold cross-validation
Rachi et al., 2023	Planning CT	Dose per segment, EQIF size	Clinical: CTV-high per unit BMI, total MU, mean oral cavity dose	SVM, KNN	Internal: accuracy evaluated with varying test splits (10–90%) and 100 random samplings
Agheli et al., 2024	Baseline CT	Radiomic: 851 features (oral mucosa); Dosiomic: 84 DVH parameters (Dmean, Dmax, Dmin, V5–V100, D5–D100)	Clinical: age, sex, BMI, stage, chemotherapy regimen	Random Forest	Internal: random 80/20 split (39/10); no external validation
Nicol et al., 2025	CT	Radiomic: 784; Dosiomic: 712; Contouromic: 576; DVH: 126. Final model used 1 clinical, 3 radiomic, 1 dosiomic	Clinical + multi-omics integration	Ridge regression, SVM, Random Forest, XGBoost, Gaussian Naïve Bayes (final: RF)	Internal cross-validation; external validation in independent dataset (separate institution)
Meng et al., 2025	Planning CT	Radiomic: 107 features; Dosiomic: 93 features (oral mucosa surface); DVH: 70 parameters (V1–V70)	Clinical: age, sex, tumor site, stage, chemotherapy, surgery, fractionated dose	Logistic regression-based model	Random 70/30 split (124/54); bootstrap (1000 iterations); no external validation
Zeng et al., 2025	Planning CT; MRI; PET-CT	Dosiometric features; Oral features (Dental calculus index, Gingival sulcus bleeding index)	Clinical: age, sex, T/N stage, chemotherapy, smoking status	Multi-classifier fusion (MCF) model, combining 15 classifiers (e.g., SVM, GNB, XGBoost)	Two-part study: a retrospective development cohort (n = 430) and a prospective intervention cohort (n = 148); a separate independent testing set was used for model evaluation

*CT* computed tomography; *OAR* organ at risk; *DVH* dose-volume histogram; *EQIF* equivalent square irradiation field; *CTV* clinical target volume; *MU* monitor unit; *BMI* body mass index; *PCA* principal component analysis; *PLR* penalized logistic regression; *RF* random forest; *SVM* support vector machine; *KNN* k-nearest neighbor; *LASSO* least absolute shrinkage and selection operator; *XGBoost* extreme gradient boosting; *RBF* radial basis function

### Model performance and calibration

Predictive performance varied substantially across studies. Dean et al. (2017) [16] reported modest discrimination, with penalized logistic regression achieving an AUC of 0.71 (SD 0.10), while random forest offered slightly lower discrimination (AUC 0.68, SD 0.08) but better calibration. Hansen et al. (2020) [17] achieved stronger performance, with LASSO and best subset models yielding AUCs of 0.797 (95% CI 0.741–0.857) and 0.808 (95% CI 0.749–0.859), respectively, in a held-out validation cohort, with robust calibration confirmed by a predictor correlation of 0.954 between models. The comparison between the performance of integrated models and their corresponding clinical or dosimetric baselines is summarized in Table 3.

**Table 3:** Comparison between baseline and multi-omic model performance

Study	Baseline predictors	Baseline AUC (Int/Ext)	Multi-omic Predictors	Multi-omic AUC (Int/Ext)	Incremental Gain?
Nicol et al., 2025	Clinical	0.63/0.56	Multi-omic	0.67/0.65	Yes (Maintained)
Hansen et al., 2020	NR	NR	PCA + Clinical	0.79/0.80	N/A
Zeng et al., 2025	NR	NR	Multi-classifier	0.90 (Test set)	Yes

*NR* not reported; *N/A* not applicable

In contrast, smaller exploratory studies reported strikingly high metrics but under fragile conditions. Rachi et al. (2023) [18] achieved mean accuracies of 0.981 (SVM) and 0.972 (KNN), yet these results were generated from unconventional validation of hundreds of predictors sets within only 61 patients, raising acute concerns of overfitting and optimistic bias. Similarly, Agheli et al. (2024) [19] reported an internal AUC of 0.917, but this was based on a training/testing split that left only ten patients in the test set, severely undermining generalizability.

More advanced pipelines revealed the tension between apparent performance and external reproducibility. Nicol et al. (2025) [20], the only study with true external validation, demonstrated the attenuation of internal gains: while its internal AUC reached 0.67, external validation in an independent cohort reduced this to 0.65 (95% CI 0.53–0.77), underscoring the gap between apparent robustness and real-world transportability. Meng et al. (2025) [21], within the CIRT setting, reported some of the highest values observed, with AUCs of 0.932 (training) and 0.959 (test) alongside accuracies of 87–91%, supported by 1,000 bootstrap iterations; however, the model’s modality-specific scope restricts broader application. Finally, Zeng et al. (2025) [22] demonstrated that oral health indices added meaningful predictive power, with their multi-classifier fusion model reaching an AUC of 0.904, accuracy of 84.9%, sensitivity of 85.3%, and specificity of 84.6% in a prospectively validated cohort of 148 patients. Collectively, discrimination metrics ranged from 0.70 to 0.96, but the most impressive values were confined to internally validated or narrowly scoped settings (Table 4).

**Table 4:** Predictive performance of radiomic/dosiomic models for oral mucositis

Study	Best-performing model	Predictors included	Performance metrics	Validation details
Dean et al., 2017	RFC (random forest) models performed better due to superior calibration and probability estimates, although they showed slightly lower discrimination (AUC). The novel OAR (MSC-PM) did not outperform the simpler OCC-PM OAR	Dose-volume metrics, dose spatial metrics, and clinical covariates. Dose-volume and spatial metrics were most strongly associated with mucositis	AUC for PLR OCC-PM,sta model: 0.71 (SD: 0.10); AUC for RFC MSC-PM,sta model: 0.68 (SD: 0.08). Overall, models showed modest to good discrimination	Internal validation with 100-iteration stratified shuffle split (80/20). No external validation performed
Hansen et al., 2020	Both LASSO and Best Subset models showed robust and highly similar performance	Treatment acceleration and PC1–PC2 were included in both models. The LASSO model also included sex and smoking status	LASSO model AUC: 0.797 (95% CI: 0.741–0.857); Best Subset model AUC: 0.808 (95% CI: 0.749–0.859). Both models demonstrated good calibration	Random 2/3–1/3 split into modeling and validation cohorts. Internal tenfold cross-validation for parameter selection
Rachi et al., 2023	SVM (support vector machine) and KNN (k-nearest neighbor)	EQIF size (cm^2^), CTV high/BMI (cm^3^/m^2^/kg), total MU, dose per segment (Gy), mean oral cavity dose (Gy)	SVM mean accuracy: 0.981 ± 0.020; KNN mean accuracy: 0.972 ± 0.033	Validation based on varying test size (10–90%) and random extractions (1–100 iterations)
Agheli et al., 2024	Random forest model	Radiomic, dosimetric, and clinical features integrated	AUC: 91.7%; Accuracy: 90%; Sensitivity: 83%; Specificity: 100%; Precision: 100%; F1-score: 91.0%	Internal validation with 80/20 random split (n = 39 training/n = 10 testing)
Nicol et al., 2025	Multi-omic random forest classifier outperformed conventional models	Radiomic, dosiomic, and clinical features	Multi-omic model – Internal AUC: 0.67 (95% CI: 0.61–0.73); External AUC: 0.65 (95% CI: 0.53–0.77). Conventional model – Internal AUC: 0.63 (95% CI: 0.56–0.69); External AUC: 0.56 (95% CI: 0.44–0.67)	Internal cross-validation and external validation on an independent cohort
Meng et al., 2025	Hybrid model combining DVH (V37 Gy[RBE]), a dosiomic feature (GLSZM_HGLZE), and a radiomic feature (GLCM_MCC)	DVH, dosiomic, and radiomic features	Training set: AUC 0.932, Accuracy 87.1%; Test set: AUC 0.959, Accuracy 90.7%	Bootstrap-based validation (1000 iterations). Hybrid model significantly outperformed single-feature models (Bonferroni-adjusted p < 0.001)
Zeng et al., 2025	The multi-classifier fusion (MCF) model, integrating all features, showed the best performance	A combination of dosimetric, clinical, and oral features. Key oral predictors were the dental calculus index and gingival sulcus bleeding index	Best Model Performance (on independent test set): AUC: 0.904; Accuracy: 84.9%; Sensitivity: 85.3%; Specificity: 84.6%	A two-part study with a retrospective development cohort (n = 430) and a prospective verification cohort (n = 148). The development cohort was used to create and evaluate the model on an independent test set

*RFC* Random forest classifier; *PLR* Penalized logistic regression; *OAR* Organ at risk; *OCC-PM* Oral cavity contour–planning margin; *MSC-PM* Mucosal surface contour–planning margin; *SD* Standard deviation; *LASSO* Least absolute shrinkage and selection operator; *PCA* Principal component analysis; *SVM* Support vector machine; *KNN* K-nearest neighbor; *EQIF* Equivalent square irradiation field; *CTV* Clinical target volume; *BMI* Body mass index; *MU* Monitor unit; *AUC* Area under the receiver operating characteristic curve; *CI* Confidence interval; *DVH* Dose–volume histogram; *GLSZM_HGLZE* Gray-level size zone matrix, high gray-level emphasis; GLCM_MCC Gray-level co-occurrence matrix, maximal correlation coefficient; *RBE* Relative biological effectiveness; *MCF* Multi-classifier fusion

### Risk of bias and applicability (PROBAST)

The overall methodological quality varied across studies, with most showing low risk of bias in the domains of participants, predictors, and outcomes, but recurrent vulnerabilities in the analysis domain. Common issues included limited external validation, small test sets, and incomplete reporting of calibration. Only one study achieved low risk across all PROBAST domains through a multicenter design with independent validation [20]. Full results of the PROBAST appraisal are presented in Table 5 and Supplementary Table 4.

**Table 5:** PROBAST—risk of bias and applicability

Study	Risk of bias				Applicability				
	Participants	Predictors	Outcome	Analysis	Participants	Predictors	Outcome	Overall RoB	Overall applicability
Dean et al., 2017	?	?	+	−	+	?	+	−	?
Hansen et al., 2020	+	+	+	?	+	+	+	?	?
Rachi et al., 2023	+	?	+	−	+	?	+	−	?
Agheli et al., 2024	+	?	+	−	+	?	+	−	?
Nicol et al., 2025	+	+	+	+	+	+	+	+	+
Meng et al., 2025	+	+	+	−	+	+	+	−	?
Zeng et al., 2025	+	+	+	?	+	+	+	?	?

Low (+); High (–); Unclear (?)

## Discussion

To our knowledge, this is the first systematic synthesis specifically focusing on the integration of radiomic and dosiomic features for OM prediction, offering a comprehensive overview of current ML strategies in HNC toxicity modeling. This systematic review assessed predictive models for radiation-induced OM that incorporated dosimetric, radiomic, dosiomic, contouromic, and host-related features with machine-learning approaches. Across seven included studies representing 2,311 patients and a range of RT modalities (IMRT/VMAT and CIRT) [16–22], several consistent messages emerge. First, dosimetric information is the most studied predictor of OM across settings [4–6]. Second, multi-omic extensions (radiomics, dosiomics, and host indices) may improve apparent discrimination in internally validated cohorts but have not yet demonstrated robust, generalizable gains on external testing [20]. Third, methodological heterogeneity and weaknesses especially in validation strategy and sample size are the principal barriers preventing translation of these models into clinical practice [23, 24].

The primacy of dose is evident across the evidence base. Every study included dose metrics (DVH parameters or spatial dose descriptors), and dosimetric components alone often produced competitive models. For example, Dean et al. [16] and Hansen et al. [17] showed that detailed dose-distribution descriptors and dose components (via PCA) were highly informative. These findings align with prior large evidence syntheses showing dose–response relationships as dominant determinants of OM risk [4–6]. Recent assessments of NTCP model generalizability based on TRIPOD criteria highlight the importance of robust validation across diverse populations [25]. In practical terms, this suggests that any novel multi-omic model should be benchmarked against a well-specified dosimetric–clinical baseline; incremental value claims must be quantified relative to that reference [26].

Radiomics, dosiomics, and other advanced predictors were selected in some final models and produced high internal AUCs in several single-center studies [19, 21]. Yet the key caveat is transportability. The multicenter study with external validation [20] distilled > 2,000 candidate features to five final predictors, but external discrimination was modest (AUC ~ 0.65), illustrating the common pattern: internally optimistic performance that attenuates on independent cohorts. Multicenter variability in radiomic features remains a major challenge, and correction procedures have been proposed to mitigate this issue [27]. This optimism is also seen in small exploratory reports, a pattern commonly described in AI-driven radiomics literature [18, 28], where very high accuracy was obtained from unconventional internal testing and few patients circumstances that raise substantial overfitting concerns. Similar issues have been widely reported in radiomics applications for other toxicities, such as xerostomia and dermatitis, where models perform well internally but lose discrimination externally [9, 29, 30].

Host-related biology predictors also deserve careful consideration. In the study by Zeng et al. [22], the inclusion of simple oral health indices significantly enhanced predictive performance when added to dosimetric variables. These results are consistent with long-standing evidence from supportive care and oncology, where poor oral hygiene and microbial imbalance have been repeatedly associated with higher OM risk [31, 32]. What distinguishes the recent contribution is not the recognition of oral health as a risk factor, but its formal integration into predictive modeling frameworks, demonstrating that such clinical and modifiable parameters can be quantitatively incorporated alongside imaging and dosimetric features. Such predictors may represent the most clinically actionable additions to OM risk models, offering tangible opportunities for toxicity mitigation. This is because they point to actions that can be taken, like improving dental health before RT, that are both possible and might work to lower toxicity.

Methodological limitations are the most important constraint on clinical translation. Our PROBAST assessment [14, 15] identified the analysis domain as the principal source of bias: frequent reliance on internal validation (random splits, cross-validation or bootstrap alone), small test sets, unstable feature selection pipelines, and incomplete reporting of calibration or uncertainty intervals. Recent efforts to develop AI-specific reporting guidelines and tools, such as TRIPOD-AI and PROBAST-AI, may help improve the rigor of future studies [33]. Only one study [20] achieved robust external validation across centers and earned a low overall RoB—yet its modest external performance underscores that external validation must be prioritized early and across diverse patient populations. This pattern mirrors broader concerns raised in TRIPOD/PROBAST guidance and in systematic reviews of radiomics: without pre-specified protocols, locked analysis pipelines, and independent validation cohorts, reported performance is likely inflated [24, 34, 35]. Moreover, calibration the agreement between predicted probabilities and observed frequencies is a critical aspect of model performance that is often overlooked, and poor calibration can limit clinical utility [36].

Although predictive performance remains a primary focus in the current literature, the clinical translation of ML models depends heavily on their interpretability [15, 33]. When evaluated against the criteria proposed by Mediouni et al. [37], current OM prediction models exhibit significant transparency gaps.

First, regarding the ability to assess individual input features: while some models identify specific contributing variables (e.g., equivalent square irradiation field or specific dosiomic textures) [18, 21], many employ complex feature reduction pipelines or ensemble algorithms that obscure the clinical weight of individual predictors [20, 23].

Second, traceability the capacity to map a specific algorithmic prediction back to a patient's underlying biological or dosimetric rationale is rarely achieved, with most pipelines functioning as 'black boxes' [24, 28].

Third, regarding the ability to assess interactions between features, current OM models largely fail to capture non-linear, synergistic relationships between clinical phenotypes and radiomic/dosiomic genotypes [25, 30]. Given that oral mucositis is a multi-factorial toxicity [1, 31], future algorithms must prioritize interpretable architectures capable of quantifying combinatorial and synergistic effects among dose, imaging features, and host biology, thereby fostering clinical trust and actionable decision-making [33, 37].

Finally, future research should shift from technical feasibility toward clinical utility. Beyond achieving high AUCs, predictive models must demonstrate that their application changes patient care, for instance, by enabling dose adaptation, improving prophylactic strategies, or guiding dental optimization before RT. Randomized or pragmatic impact studies may be required to show that model-informed interventions reduce OM severity or treatment interruptions, ultimately improving patient-centered outcomes [23, 24]. The COVID-19 pandemic highlighted the risks of rapid deployment of poorly validated prediction models, and the same principles apply to OM prediction models [38]. Particular attention should be given to actionable predictors, such as oral health indices, which are clinically modifiable and thus hold promise for immediate translational impact [3, 32]. At the same time, collaborative data-sharing frameworks, including federated learning approaches, could help overcome privacy and institutional barriers while enabling the construction of larger, more diverse, and methodologically robust multi-omic datasets for toxicity prediction [39].

Several limitations of this systematic review must be acknowledged, most notably the substantial heterogeneity in oral mucositis (OM) endpoint definitions across the included literature. While initial assessments might suggest comparability, closer inspection shows that variations between different versions of the Common Terminology Criteria for Adverse Events (CTCAE v2.0 to v5.0) and the use of the DAHANCA scale introduce important grading differences that complicate direct comparison. Furthermore, the clinical thresholds used to define severe OM varied considerably: while the majority of studies focused on Grade ≥ 3, others adopted Grade ≥ 2 requiring adaptive radiotherapy as the primary outcome. Such disparities in scoring systems and severity thresholds preclude the statistical pooling of performance metrics such as sensitivity and specificity and render a formal meta-analysis or pooled performance estimates unfeasible.

## Conclusions

In conclusion, current evidence for multi-omic machine learning models predicting radiation-induced OM remains exploratory rather than clinically ready. Although these models often demonstrate apparent high discrimination in internally validated series, this performance frequently fails to translate to external cohorts. To move toward clinically useful tools, the field must converge on harmonized outcome definitions, enforce pre-registered methodological rigor, prioritize external and prospective testing, and emphasize clinically actionable predictors and impact evaluation. Only through these steps can predictive modeling progress from technical feasibility to meaningful improvements in patient care and treatment tolerability in head and neck oncology.

## Supplementary Information

Below is the link to the electronic supplementary material.

Supplementary file1 (DOCX 25 KB)

## Data Availability

No datasets were generated or analysed during the current study.
