# Supplementary material for: Radiomic and dosiomic machine learning models for predicting radiation-induced oral mucositis in head and neck cancer: a systematic review
Source: Clin Transl Oncol. 2026 Jun 8;28(11):5492–503. doi: 10.1007/s12094-026-04427-0 (PMC13694293; doi:10.1007/s12094-026-04427-0)
Supplement: Supplementary file 1 [file 12094_2026_4427_MOESM1_ESM.docx]

Supplementary Table 1. Full Search Strategies for All Databases.

| **Database** | **Search Query Used** | **Notes** |
| --- | --- | --- |
| **PubMed** | (("radiomics"[Title/Abstract] OR "radiomic"[Title/Abstract] OR "texture analysis"[Title/Abstract] OR "image-based prediction"[Title/Abstract] OR "prediction model"[Title/Abstract] OR "machine learning"[Title/Abstract]) AND ("mucositis"[Title/Abstract] OR "oral mucositis"[Title/Abstract] OR "oral toxicity"[Title/Abstract] OR "radiation-induced toxicity"[Title/Abstract] OR "complication"[Title/Abstract]) AND ("head and neck cancer"[Title/Abstract] OR "nasopharyngeal carcinoma"[Title/Abstract] OR "oral cancer"[Title/Abstract] OR "radiotherapy"[Title/Abstract] OR "radiation therapy"[Title/Abstract])) | Search conducted on 31/07/2025; no language or time restrictions applied; filters: humans, articles only. |
| **EMBASE** | ('radiomics'/exp OR radiomic*:ti,ab OR 'texture analysis':ti,ab OR 'prediction model':ti,ab OR 'image-based prediction':ti,ab OR 'machine learning':ti,ab) AND ('oral mucositis'/exp OR mucositis:ti,ab OR 'oral toxicity':ti,ab OR 'radiation-induced toxicity':ti,ab OR complication*:ti,ab) AND ('head and neck cancer'/exp OR 'nasopharyngeal carcinoma'/exp OR 'oral cancer'/exp OR 'radiotherapy'/exp OR 'radiation therapy':ti,ab) | Search conducted on 31/07/2025; Emtree terms and keywords combined; filters: articles only, no language restriction. |
| **Scopus** | TITLE-ABS-KEY ( radiomics OR radiomic OR "texture analysis" OR "prediction model" OR "image-based prediction" OR "machine learning" ) AND TITLE-ABS-KEY ( mucositis OR "oral mucositis" OR "oral toxicity" OR "radiation-induced toxicity" OR complication ) AND TITLE-ABS-KEY ( "head and neck cancer" OR "nasopharyngeal carcinoma" OR "oral cancer" OR radiotherapy OR "radiation therapy" ) AND ( LIMIT-TO ( DOCTYPE,"ar" ) ) | Search conducted on 31/07/2025; filters: articles only, no language restriction. |
| **Web of Science (WoS)** | TS=( radiomics OR radiomic OR "texture analysis" OR "prediction model" OR "image-based prediction" OR "machine learning" ) AND TS=( mucositis OR "oral mucositis" OR "oral toxicity" OR "radiation-induced toxicity" OR complication ) AND TS=( "head and neck cancer" OR "nasopharyngeal carcinoma" OR "oral cancer" OR radiotherapy OR "radiation therapy" ) | Search conducted on 31/07/2025; filters: articles only, no language restriction. |

Supplementary Table 2. Full-text studies excluded with reasons

| **Study** | **Study aim** | **Reason for exclusion** |
| --- | --- | --- |
| Dean et al., 2016 | To develop NTCP models using spatial dose metrics. | Duplicated cohort with Dean et al., 2017. |
| Stevenson et al., 2018 | To identify predictors of wound complications. | Population not HNC; extremity sarcoma. |
| Hansen et al., 2020 | To predict OM using clinical and patient-reported factors. | No radiomic or dosiomic features used. |
| Pardo-Montero et al., 2021 | To model mucosal toxicity using cell dynamics. | Did not perform predictive modeling. |
| Poolakkad et al., 2021 | To predict chemotherapy-induced mucositis. | Not radiation-induced OM; no imaging data. |
| Thukral et al., 2023 | To predict OM using thermographic imaging and AI. | Used thermography, not radiomic/dosiomic data. |
| Padannayil et al., 2023 | To reduce radiation dermatitis using ML planning. | Outcome was dermatitis, not mucositis. |
| Dong et al., 2023 | Develop a multimodal model (clinical + dosimetric + radiomic) to predict severe oral mucositis in NPC. | Duplicate cohort with Nicol et al., 2025. |
| Kauark-Fontes et al., 2025 | To predict OM risk using clinical variables. | Clinical data only; no imaging features. |
| Li et al., 2025 | To predict severe OM using clinical data. | Based only on clinical predictors. |
| Liang et al. 2025 | To identify clinical risk factors for OM. | Clinical model only; no imaging data. |

*AI, artificial intelligence; DVH, dose–volume histogram; HNC, head and neck cancer; IMPT, intensity-modulated proton therapy; ML, machine learning; NTCP, normal tissue complication probability; OM, oral mucositis; RT, radiotherapy.*

| **Study** | **Study goal** | **Imaging & data used** | **Key predictors retained in final model** | **Main results / take-home message** | **Stated limitations (author-reported)** | **Reviewer notes** |
| --- | --- | --- | --- | --- | --- | --- |
| **Dean et al., 2017** | To develop an NTCP model for severe acute mucositis using a novel oral mucosal surface OAR. | Retrospective data from six clinical trials, including planning CTs, DVHs, and clinical data. | Dose-volume and dose spatial metrics were the strongest predictors. | Traditional oral cavity OAR models performed as well as those using the novel mucosal surface OAR. | Lack of external validation; handling of missing data may have introduced bias. | Well-designed NTCP study with innovative OAR concept. Generalizability limited by absence of external validation and potential bias. |
| **Hansen et al., 2020** | To develop robust models predicting severe radiation-induced mucositis in a large HNC cohort. | Planning CT scans; DVH metrics transformed using PCA. | Treatment acceleration and PC1–PC2; LASSO model also included sex and smoking status. | Both LASSO and Best Subset models performed well (AUC ~0.80) and demonstrated good calibration. | Lack of genomic/microbiomic data; external validation recommended. | Strong dosiomic study with robust methodology (PCA use + independent validation cohort). Still limited by absence of external validation. |
| **Rachi et al., 2023** | To predict mucositis ≥ Grade 2 requiring adaptive radiotherapy (ART). | DICOM RT plan and structure data. | EQIF size, CTV high/BMI, total MU, dose per segment, mean oral cavity dose. | High predictive accuracy with SVM (0.981) and KNN (0.972). Mean oral cavity dose was the strongest predictor. | Small single-center dataset; lack of external validation. | Useful proof-of-concept for hybrid modeling. Promising but overfitting risk high without external validation. |
| **Agheli et al., 2024** | To enable early prediction of RIOM in HNC patients. | Baseline CT images; dosimetric + clinical data; oral mucosa manually contoured. | Top 10 radiomics, 5 dosimetric, and 5 clinical features selected via mRMR. | Integrated model (radiomics + dosimetric + clinical) achieved highest AUC (91.7%). Radiomics-only model also strong (AUC 87.0%). | Single-center; small cohort; lack of external validation. | Promising integrated multi-feature model. Generalizability limited by sample size and absence of multicenter validation. |
| **Nicol et al., 2025** | To develop and externally validate a multi-omic model for severe OM in NPC. | Pre-treatment CT, dosimetric data, clinical, DVH, radiomic, dosiomic, and contouromic features. | Final Random Forest model: 1 clinical, 3 radiomic, 1 dosiomic. | Multi-omic model outperformed conventional model (External AUC 0.65). | Authors suggested need for multi-center validation; limitations not explicitly detailed. | Significant as one of the few externally validated models. Predictive value modest, but important step toward clinical applicability. |
| **Meng et al., 2025** | To develop the first NTCP model for Grade >2 acute OM in HNC patients treated with CIRT. | Planning CT; multi-omic data (DVH, radiomic, dosiomic, clinical). | Hybrid model: DVH (V37 Gy[RBE]), Radiomics_GLCM_MCC, Dosiomics_GLSZM_HGLZE. | Hybrid model achieved very high accuracy (Test AUC 0.959; Accuracy 90.7%). Outperformed all single-feature models. | Retrospective; no external validation; specific to CIRT; anatomical changes during RT not considered. | Landmark study for CIRT. Very strong hybrid model but findings not generalizable beyond carbon-ion therapy. |
| **Zeng et al., 2025** | To develop an AI model predicting severe RIOM and test oral health factors as risk predictors in NPC. | Retrospective planning CTs and clinical data; DVHs + oral indices (OCI, DCI). | Mean oral cavity dose, OCI, DCI. | Best model achieved AUC 0.841 in validation cohort. Oral health indices improved prediction. | Single-center retrospective study; validation cohort not external. | Novel and clinically relevant approach introducing oral health features. Independent validation cohort is a strength, but external validation still needed. |

Supplementary Table 3. Summary of findings across included studies

*AOM, acute oral mucositis; ART, adaptive radiotherapy; AUC, area under the curve; BMI, body mass index; CIRT, carbon-ion radiotherapy; CRT, chemoradiotherapy; CT, computed tomography; CTCAE, Common Terminology Criteria for Adverse Events; CTV, clinical target volume; DCI, Dental Calculus Index; DICOM, Digital Imaging and Communications in Medicine; DVH, dose–volume histogram; EQIF, equivalent square irradiation field; Gy, Gray; IMRT, intensity-modulated radiotherapy; KNN, k-nearest neighbor; mRMR, minimum redundancy maximum relevance; MU, monitor units; NPC, nasopharyngeal carcinoma; NTCP, normal tissue complication probability; OAR, organ at risk; OCI, Oral Cleanliness Index; PCA, principal component analysis; RIOM, radiation-induced oral mucositis; RT, radiotherapy; SVM, support vector machine; VMAT, volumetric modulated arc therapy.*

Supplementary Table 4. PROBAST detailed domain-level ratings for included studies

| **Study** | **Participants** | **Predictors** | **Outcome** | **Analysis** | **Overall RoB** | **Overall Applicability** |
| --- | --- | --- | --- | --- | --- | --- |
| **Dean et al., 2017** | Unclear (?) – Cohort of 179 patients from six clinical trials; missing data handling strategy may have introduced bias. | Unclear (?) – Key predictors such as smoking and alcohol use were not available, potentially reducing validity. | Low risk (+) – Severe OM (≥ grade 3) clearly defined and consistently assessed with CTCAE. | High risk (–) – Internal nested cross-validation only; no external validation performed. | High risk (–) – Main limitation is lack of external validation and incomplete predictor data. | Unclear (?) – Applicability uncertain due to absence of relevant predictors and reliance on novel OAR definition. |
| **Hansen et al., 2020** | Low risk (+) – Large, prospective, representative HNC cohort (n=802). | Low risk (+) – Well-defined predictors (DVH via PCA, clinical covariates). | Low risk (+) – Severe OM (≥ grade 3) measured with DAHANCA scale (equivalent to CTCAE). | Unclear (?) – Validation performed on a sub-cohort (1/3) rather than independent external data. | Unclear (?) – Main limitation is lack of truly external validation. | Low risk (+) – Highly applicable as data came from a prospective clinical practice cohort. |
| **Rachi et al., 2023** | Low risk (+) – Well-defined single-center cohort (n=61). | Unclear (?) – Some predictors (BMI, mean oral dose) collected only at baseline, not longitudinally. | Low risk (+) – OM requiring ART, assessed with CTCAE v5.0. | High risk (–) – Small sample; extensive feature engineering (671 lists); only internal validation; strong risk of overfitting. | High risk (–) – Overall high due to small sample size and lack of robust validation. | Unclear (?) – Applicability limited by small, single-center dataset. |
| **Agheli et al., 2024** | Low risk (+) – Prospective, single-center cohort with clear inclusion/exclusion (n=49). | Low risk (+) – Radiomic, dosimetric, and clinical features clearly defined; MRI harmonization applied. | Low risk (+) – Severe OM (≥ grade 3) assessed with CTCAE v5.0. | High risk (–) – Internal validation only (80/20 split, n=10 test set); no external validation. | High risk (–) – Lack of external validation reduces generalizability. | Unclear (?) – Applicability uncertain given single-center design and small cohort. |
| **Nicol et al., 2025** | Low risk (+) – Large multicenter cohort (n=464) with independent external validation cohort. | Low risk (+) – Broad, well-defined predictors (clinical, radiomic, dosiomic, contouromic). | Low risk (+) – Severe OM (≥ grade 3) consistently defined with CTCAE v5.0. | Low risk (+) – Both internal cross-validation and external validation performed. | Low risk (+) – Strong design, multicenter, external validation. | Low risk (+) – High applicability due to multicenter design and external testing. |
| **Meng et al., 2025** | Low risk (+) – Retrospective but well-defined cohort (n=178). | Low risk (+) – Multi-omic predictors (DVH, radiomic, dosiomic). | Low risk (+) – OM > grade 2 defined with CTCAE v4.0. | High risk (–) – Retrospective single-center; internal validation (bootstrap, split 70/30); no external validation. | High risk (–) – Lack of external validation limits generalizability. | Unclear (?) – Applicability limited since model was developed specifically for CIRT. |
| **Zeng et al., 2025** | Low risk (+) – Large single-center cohort (n=578) focused on NPC. | Low risk (+) – Clinical, dosimetric, and novel oral features (OCI, DCI) included. | Low risk (+) – Severe OM (≥ grade 3) assessed with CTCAE v5.0. | Unclear (?) – Independent internal validation cohort (n=148) but from the same center; no multicenter external validation. | Unclear (?) – Lack of external multicenter validation limits generalizability. | Unclear (?) – Applicability uncertain since results not tested across populations with different clinical/genomic profiles. |

*Oral mucositis, OM; Common Terminology Criteria for Adverse Events, CTCAE; Danish Head and Neck Cancer Group toxicity scale, DAHANCA; Organ at risk, OAR; Principal Component Analysis, PCA; Adaptive Radiotherapy, ART; Carbon-ion radiotherapy, CIRT; Oral Cleanliness Index, OCI; Dental Calculus Index, DCI; Low (+); High (–); Unclear (?).*
